# Impact of errors in spot size and spot position in robustly optimized pencil beam scanning proton‐based stereotactic body radiation therapy (SBRT) lung plans

**DOI:** 10.1002/acm2.13293

**Published:** 2021-06-07

**Authors:** Suresh Rana, Anatoly B. Rosenfeld

**Affiliations:** ^1^ Department of Medical Physics The Oklahoma Proton Center Oklahoma City Oklahoma USA; ^2^ Department of Radiation Oncology Miami Cancer Institute Baptist Health South Florida Miami Florida USA; ^3^ Department of Radiation Oncology Herbert Wertheim College of Medicine Florida International University Miami Florida USA; ^4^ Centre for Medical Radiation Physics (CMRP) University of Wollongong Wollongong New South Wales Australia

**Keywords:** lung cancer, Monte Carlo, proton therapy, robustness, robust optimization, SBRT, spot position, spot size

## Abstract

**Purpose:**

The purpose of the current study was threefold: (a) investigate the impact of the variations (errors) in spot sizes in robustly optimized pencil beam scanning (PBS) proton‐based stereotactic body radiation therapy (SBRT) lung plans, (b) evaluate the impact of spot sizes and position errors simultaneously, and (c) assess the overall effect of spot size and position errors occurring simultaneously in conjunction with either setup or range errors.

**Methods:**

In this retrospective study, computed tomography (CT) data set of five lung patients was selected. Treatment plans were regenerated for a total dose of 5000 cGy(RBE) in 5 fractions using a single‐field optimization (SFO) technique. Monte Carlo was used for the plan optimization and final dose calculations. Nominal plans were normalized such that 99% of the clinical target volume (CTV) received the prescription dose. The analysis was divided into three groups. *Group 1*: The increasing and decreasing spot sizes were evaluated for ±10%, ±15%, and ±20% errors. *Group 2*: Errors in spot size and spot positions were evaluated simultaneously (spot size: ±10%; spot position: ±1 and ±2 mm). *Group 3*: Simulated plans from *Group 2* were evaluated for the setup (±5 mm) and range (±3.5%) errors.

**Results:**

*Group 1*: For the spot size errors of ±10%, the average reduction in D_99%_ for −10% and +10% errors was 0.7% and 1.1%, respectively. For −15% and +15% spot size errors, the average reduction in D_99%_ was 1.4% and 1.9%, respectively. The average reduction in D_99%_ was 2.1% for −20% error and 2.8% for +20% error. The hot spot evaluation showed that, for the same magnitude of error, the decreasing spot sizes resulted in a positive difference (hotter plan) when compared with the increasing spot sizes. *Group 2*: For a 10% increase in spot size in conjunction with a −1 mm (+1 mm) shift in spot position, the average reduction in D_99%_ was 1.5% (1.8%). For a 10% decrease in spot size in conjunction with a −1 mm (+1 mm) shift in spot position, the reduction in D_99%_ was 0.8% (0.9%). For the spot size errors of ±10% and spot position errors of ±2 mm, the average reduction in D_99%_ was 2.4%. *Group 3*: Based on the results from 160 plans (4 plans for spot size [±10%] and position [±1 mm] errors × 8 scenarios × 5 patients), the average D_99%_ was 4748 cGy(RBE) with the average reduction of 5.0%. The isocentric shift in the superior–inferior direction yielded the least homogenous dose distributions inside the target volume.

**Conclusion:**

The increasing spot sizes resulted in decreased target coverage and dose homogeneity. Similarly, the decreasing spot sizes led to a loss of target coverage, overdosage, and degradation of dose homogeneity. The addition of spot size and position errors to plan robustness parameters (setup and range uncertainties) increased the target coverage loss and decreased the dose homogeneity.

## 1 INTRODUCTION

In pencil beam scanning (PBS) proton delivery, the accuracy of the size and position of a pencil proton beam is very critical to minimize the discrepancies between the delivered and computed doses. Spot sizes on the proton beam delivery system can be affected by the fluctuations in the beam extraction and transport systems.^1^ Additionally, the presence of different scattering materials in the nozzle,^1^ as well as the air gap between the range shifter and patient, can have an impact on the spot size.^2^ Similarly, the positioning of the spots can be affected by the fluctuations in the steering magnetic fields.^3^, ^4^ Hence, the variations in the delivered spot sizes and positions could lead to perturbation of dose distributions impacting the quality of the treatment plan delivered to the patient.^1^, ^2^, ^3^, ^4^, ^5^, ^6^, ^7^

In order to minimize the discrepancies between the computed and delivered dose distributions in PBS proton therapy, tolerance levels are proposed for the spot size and position errors. Parodi et al.^1^ evaluated the impact of spot size on spherical phantom plans and proposed the tolerance of ±50%. Chanrion et al.^5^ studied the variations in spot sizes on prostate and skull‐base patients and reported negligible to moderate changes in dose distributions when spot sizes are changed by ⩽25%. Lin et al.^8^ performed a study on 28 patients of different disease sites (breast, sarcoma, central nervous system, pediatric, gastrointestinal, genitourinary, and gynecological). Based on their gamma analysis, the spot size tolerance of ±10% was proposed.^8^ Kraan and colleagues^7^ demonstrated that the variation in spot size is patient and spot width dependent. Their study^7^ included seven patients of different disease sites (pelvis, chest wall, rectum, chordoma, cardiac, retro‐peritoneal, and sarcoma) and a phantom. If in‐air one sigma (σ) of a pencil beam is 2.5 mm, the tolerance is ±25%.^7^ Similarly, for σ of 5 and 10 mm, the proposed tolerances are ±25% and ±10%, respectively.^7^ For the spot position errors, the tolerance of ±1 mm has been reported by the investigators.^4^, ^8^, ^9^, ^10^ Recently, the AAPM TG224 report^11^ recommended the tolerance of ±10% for the spot size and ±1 mm for the spot position.

Previous publications^1^, ^2^, ^3^, ^4^, ^5^, ^6^, ^7^, ^8^, ^9^ have reported the variations in spot size and position in the phantoms and disease sites but not for the lung. For PBS lung cancer treatment, the accuracy of the dose calculation algorithm in predicting spot size and dose distributions becomes more critical due to varying tissue densities in the proton beam path. In commercial proton treatment planning systems (TPS), Monte Carlo algorithms have been shown to be more accurate in estimating spot sizes than analytical pencil beam algorithms.^12^, ^13^ A growing number of publications^14^, ^15^, ^16^, ^17^ have now recommended using the Monte Carlo algorithm for the dose calculations in PBS lung cancer. Recently, robust optimization^14^, ^18^ feature has been made available in the clinical environment, whereas previous studies^1^, ^5^, ^6^, ^7^, ^8^ did not address the impact of variation in spot size on robustly optimized clinical plans. It is essential to understand the effects of errors in spot sizes on the Monte Carlo algorithm‐based robustly optimized PBS lung cancer plans. Additionally, none of the previous studies^1^, ^2^, ^3^, ^4^, ^5^, ^6^, ^7^, ^8^, ^9^ have studied the impact of the errors in spot sizes and positions simultaneously. In the current study, we aim to answer the following questions regarding the robustly optimized PBS proton‐based stereotactic body radiation therapy (SBRT) lung plans:

1. What is the dosimetric impact of spot size errors of ±10%, ±15%, and ±20%?
2. What is the dosimetric impact of spot size and position errors occurring simultaneously? The simultaneous evaluation is performed by combining spot size and position errors (spot size: ±10% and spot position: ±1 and ±2 mm)?
3. What are the overall effect of spot size (±10%) and position (±1 mm) errors in conjunction with either setup (±5 mm) or range (±3.5%) errors?

## 2 MATERIALS AND METHODS

### 2.A Contouring and treatment planning

In this retrospective study, PBS lung plans were replanned on the computed tomography (CT) data set of five lung patients. The clinical target volume (CTV) ranged from 24.27 to 63.24 cc. The CTV was created by an isotropic margin of 5 mm around the internal gross tumor volume (IGTV). The IGTV was obtained based on the four‐dimensional computed tomography (4DCT) images. For proton planning, the average intensity projection CT was utilized.

RayStation TPS (Version 9B; RaySearch Laboratories, Stockholm, Sweden) was used for treatment planning. The proton beam model is based on the IBA ProteusPLUS proton therapy system with a PBS dedicated nozzle (Ion Beam Applications, Louvain‐la‐Neuve, Belgium).^19^, ^20^ The in‐air one sigma (σ) for 226.5 MeV at the isocenter is ~3 mm.^19^ For each patient, a nominal plan was regenerated for a total dose of 5000 cGy(RBE) in 5 fractions using an average RBE of 1.1. Treatment plans were robustly optimized using a single‐field optimization (SFO) technique. The Monte Carlo algorithm (10 000 ions/spot) was utilized for the robust optimization. The robustness (range uncertainty = ±3.5% and setup error = ±5 mm) was applied on the CTV such that its 99% of the relative volume receives at least the prescription dose (5000 cGy(RBE)). Based on the input values of robustness parameters, RayStation optimized each plan for a total of 21 scenarios. The final dose calculations were performed using the Monte Carlo (grid size: 2 mm; statistical uncertainty = 0.5%). This was followed by the creation of a volumetric repainting plan with five paintings in an alternating order.^21^, ^22^ The resulting plan was then normalized such that the CTV D_99%_ = 5000 cGy(RBE). The final nominal plan was denoted as *D(0%, 0* mm*)*, which means 0% error in spot size and 0‐mm error in spot position.

### 2.B Spot size errors simulation

In order to simulate the spot size errors of ±10%, ±15%, and ±20%, additional six beam models were generated. These were simulated by scaling the spot profiles in the nominal beam model. In the simulated beam models, absolute dose output and integrated depth doses (IDDs) remained identical as in the nominal beam model.

### 2.C Dose calculations for spot size errors only

The spot size errors calculation was performed by recomputing *D(0%, 0* mm*)* plan using the simulated beam models (±10%, ±15%, and ±20%). For instance, if *D(0%, 0* mm*)* plan was recomputed for the spot size error of +10% and spot position error of 0 mm, the resulting plan was denoted as *D(+10%, 0* mm*)*. Similarly, for −20% spot size and 0‐mm spot position errors, the plan was denoted as *D(−20%, 0* mm*)*. Dose recomputations were performed using the Monte Carlo algorithm without plan reoptimization.

### 2.D Spot position errors simulation

The *D(0%, 0* mm*)* plan containing the spot position information was exported from the TPS to a local computer. Then spot positions in the treatment plan were varied systematically by −1 and +1 mm, thus resulting in two simulated plans, *D(0%,* *−1* mm*)* and *D(0%, +1* mm*)*, respectively. This process was repeated for the systematic shift of spot positions by ±2 mm to generate *D(0%,* *−2* mm*)* and *D(0%, +2* mm*)* plans. The simulation of spot position errors was performed using an in‐house developed MatLab code (Version R2019b; MathWorks, Natick, MA, USA).

### 2.E Dose calculation for the spot size and position errors occurring simultaneously

For each patient, simulated plans for spot positions (*as described in* *Section* *2D*) were imported back into RayStation TPS. The *D(0%,* *−1* mm*)* plan was recomputed using Monte Carlo algorithm (without reoptimization) for the spot size errors of −10% and +10%, resulting in *D(−10%, −1* mm*)* and *D(+10%,* *−1* mm*)* plans, respectively. The *D(0%, +1* mm*)* plan was recomputed using Monte Carlo algorithm (without reoptimization) for the spot size errors of −10% and +10%, resulting in *D(−10%, +1* mm*)* and *D(+10%, +1* mm*)* plans, respectively. Similarly, the *D(0%,* *−2* mm*)* and *D(0%, +2* mm*)* plans were recomputed using Monte Carlo algorithm (without reoptimization) for ±10% spot size errors.

### 2.F Robustness

The *D(±10%, ±1* mm*)* plans were evaluated for a total of eight scenarios. The setup uncertainty was simulated by a 5‐mm isocenter shift in the left–right, superior–inferior, and anterior–posterior directions of the patient resulting in six scenarios. The range uncertainty was evaluated for two scenarios (±3.5%).

### 2.G Analysis

The analysis was divided into three groups. The first group (*Group 1*) consisted of plans simulated for spot size errors only, as described in Section 2.C. The second group (*Group 2*) included the plans that were simulated for spot size and position errors occurring simultaneously. The simulated plans in the *Group 2* are described in Section 2.E. Finally, the third group (*Group 3*) included the evaluation of *D(±10%, ±1* mm*)* plans for setup (±5 mm) and range (±3.5%) uncertainties as described in Section 2.F.

The difference (∆) at a dosimetric metric (e.g., D_99%_) between simulated and nominal plans was calculated using Eq. (1).

(1) $$\Delta \;=\;\frac{\left(D_{x\%}^{\mathrm{Simulated}}‐D_{x\%}^{\mathrm{Nominal}}\right)}{D_{x\%}^{\mathrm{Nominal}}}\times 100$$

$D_{x\%}^{\mathrm{Nominal}}$ = result for *x* metric (e.g., 99) in the nominal plan.

$D_{x\%}^{\mathrm{Simulated}}$ = result for *x* metric in a simulated plan.

The difference was averaged (Δ_avg_) over five patients.

(2) $$\Delta_{avg.}\;=\;\frac{1}{5}\sum_{i=1}^{n=5}\Delta_{i}$$

The CTV dose homogeneity index (HI) was evaluated using Eq. (3), as shown below:

(3) $$HI\;=\;\frac{\left(D_{1\%}‐D_{99\%}\right)}{\mathrm{Rx}}$$

where *Rx* is the prescription dose (5000 cGy(RBE)). Based on Eq. (3), the HI value of 0 is considered an ideal HI result.

## 3 RESULTS

### 3.A Group 1: Spot size errors

**Fig. 1:**
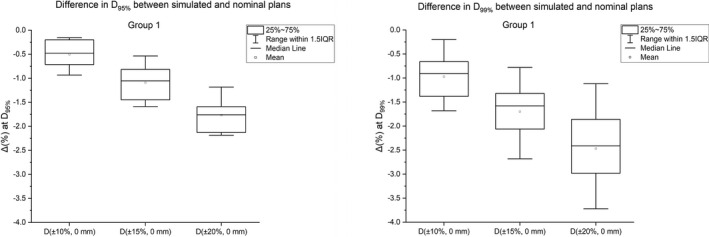
The average difference in clinical target volume D_95%_ (left panel) and D_99%_ (right panel) between simulated plans in Group 1 and nominal plans for the spot size errors (±10%, ±15%, and ±20%). The results are averaged over all five patients.

The spot size errors resulted in a loss of the target coverage (Fig. 1). The reduction in target coverage increased as the magnitude of spot size error was increased.

D_95%_: The Δ_avg_. at D_95%_ for −10% and +10% errors were −0.4% and −0.6%, respectively. The Δ_avg_. at D_95%_ was −1.0% for ±15% errors. The Δ_avg_. at D_95%_ was −1.6% for ±20% errors.

D_99%_: The Δ_avg_. at D_99%_ for −10% and +10% errors were −0.7% and −1.1%, respectively. The Δ_avg_. at D_99%_ was −1.4% for −15% error and −1.9% for +15% error. The Δ_avg_. at D_99%_ was −2.1% for −20% error and −2.8% for +20% error.

HI: On average, the difference in HI results between nominal *D(0%, 0* mm*)* and simulated plans for ±10% errors (*D(+10%, 0* mm*)* and *D(−10%, 0* mm*)*) was 0.01. For ±15% and ±20% spot size errors, decreasing spot sizes resulted in less homogeneous plans compared with increasing spot sizes. Specifically, for the spot size errors of ±15%, it was found that the average difference in HI was worse for *D(−15%, 0* mm*)* plan (0.03) than *D(+15%, 0* mm*)* plan (0.01) when their results were compared against *D(0%, 0* mm*)* plan. A similar trend was observed for the spot size errors of ±20%, with the average difference in HI being worse for *D(−20%, 0* mm*)* plan (0.04) than *D(+20%, 0* mm*)* plan (0.02) when their results were compared against *D(0%, 0* mm*)* plan.

### 3.B Group 2: Spot size and position errors occurring simultaneously

**Fig. 2:**
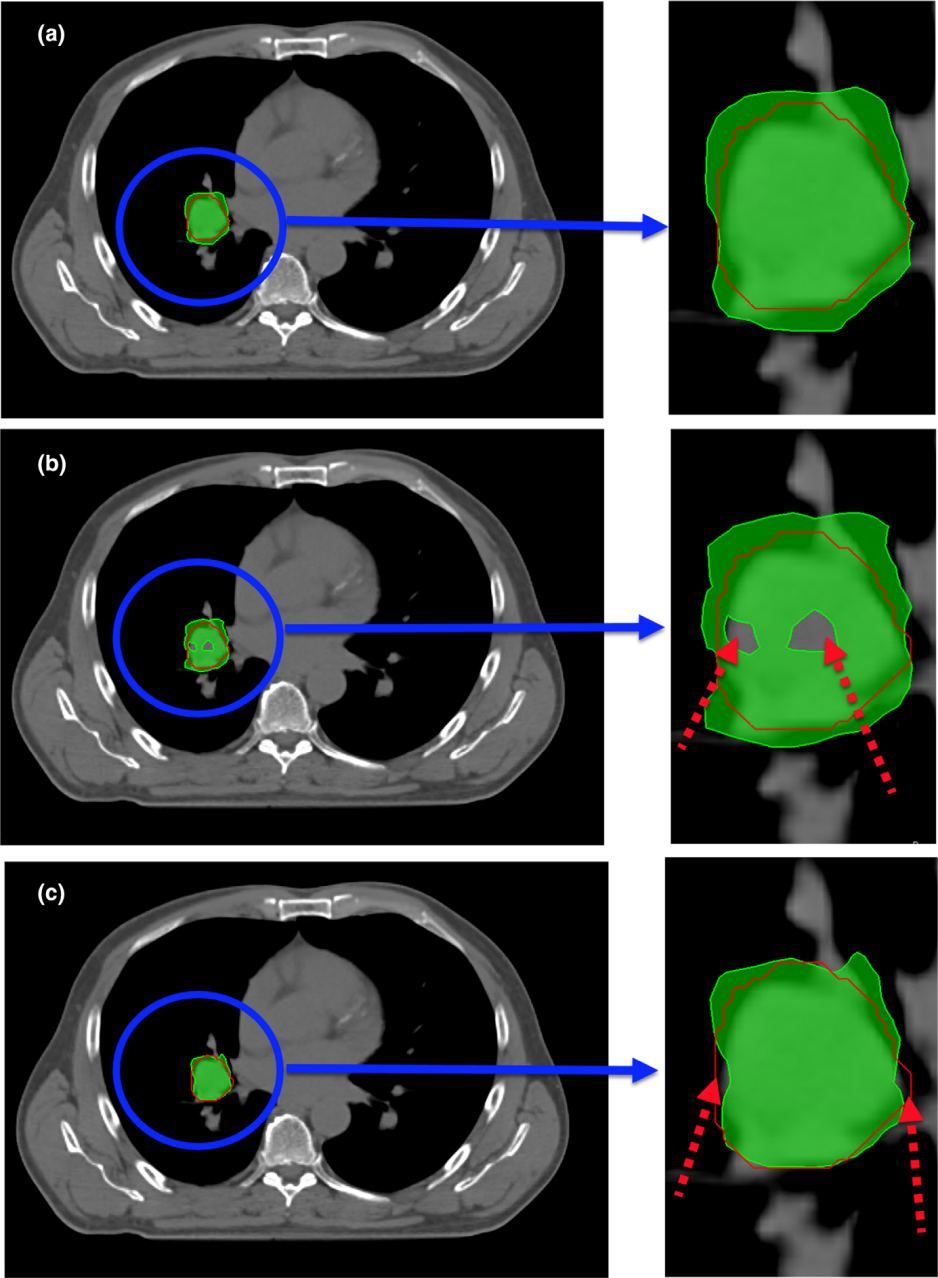
Dose distributions in an example patient: (a) nominal plan, (b) simulated plan for the decreasing spot size (−10%) and spot position (+1 mm) evaluated simultaneously; (c) simulated plan for the increasing spot size (+10%) and spot position (+1 mm) evaluated simultaneously. The loss of target coverage in the simulated plans is shown by the red arrows on the right panel.

**Fig. 3:**
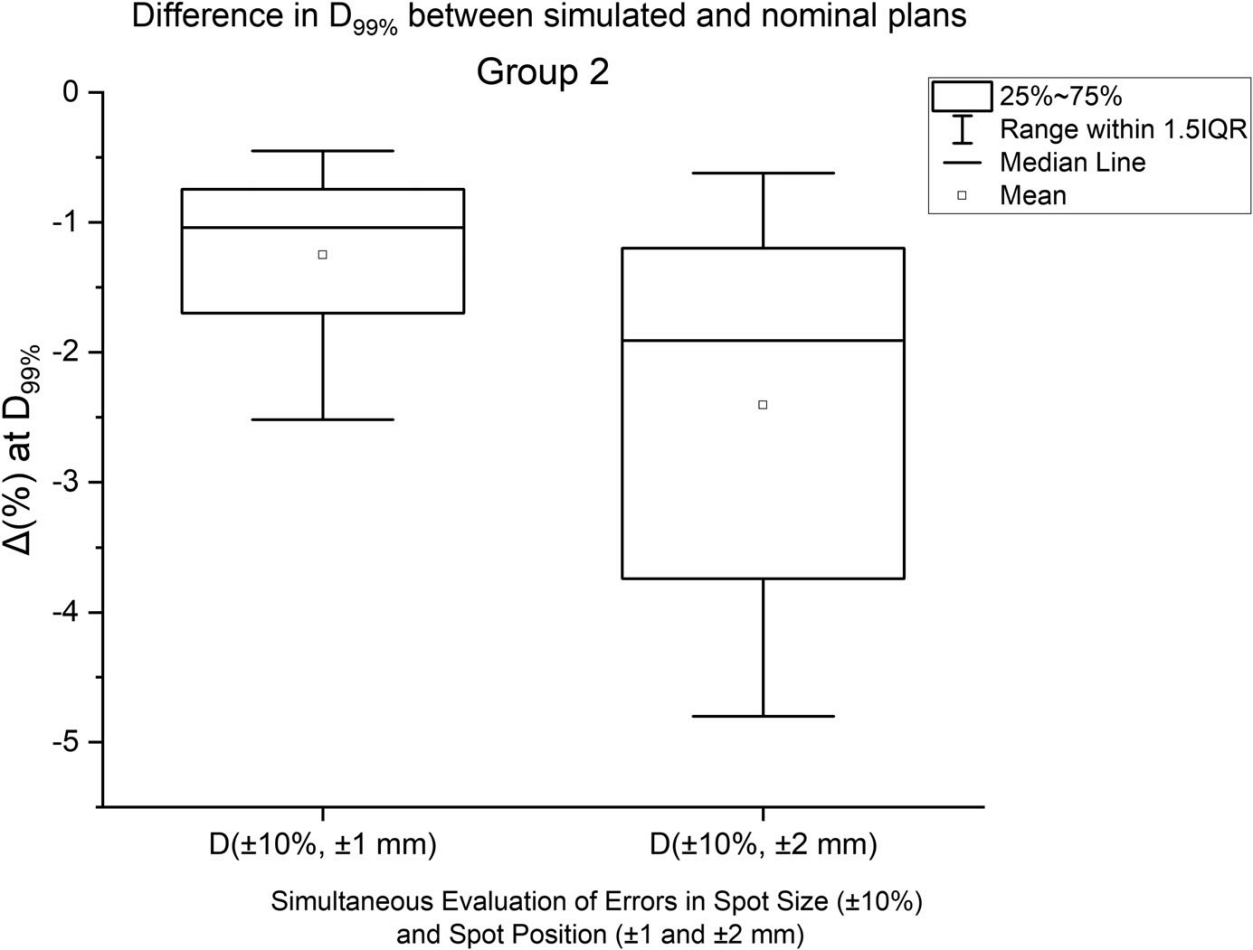
The average difference in clinical target volume D_99%_ between simulated plans in Group 2 and nominal plans for the spot size errors (±10%) in conjunction with spot position errors (±1 and ±2 mm). The results are averaged over all five patients.

Figure 2 illustrates the dose distributions in an example patient for the nominal plan and simulated plan for the combined errors of the decreasing (increasing) spot size −10% (+10%) and spot position (+1 mm). Figure 3 shows the reduction in target coverage between the nominal plan and simulated plan for the spot size errors (±10%) and spot position errors (±1 mm). For a 10% increase in spot size and ±1‐mm shift in spot position, the Δ_avg_. at D_99%_ was −1.5% for *D(+10%,* *−1* mm*)* plan and −1.8% for *D(+10%, +1* mm*)* plan. For a 10% decrease in spot size and ±1‐mm shift in spot position, the Δ_avg_. at D_99%_ was −0.8% for *D(−10%, −1* mm*)* plan and −0.9% for *D(−10%, +1* mm*)* plan. Figure 3 also exhibits the results from *D(±10%, ±2* mm*)* plans (*n* = 20) for the spot size errors of ±10% and spot position errors of ±2 mm. For *D(±10%, ±2* mm*)* plans, the Δ_avg_. at D_99%_ was −2.4%.

### 3.C Group 3: Setup and range errors

**Fig. 4:**
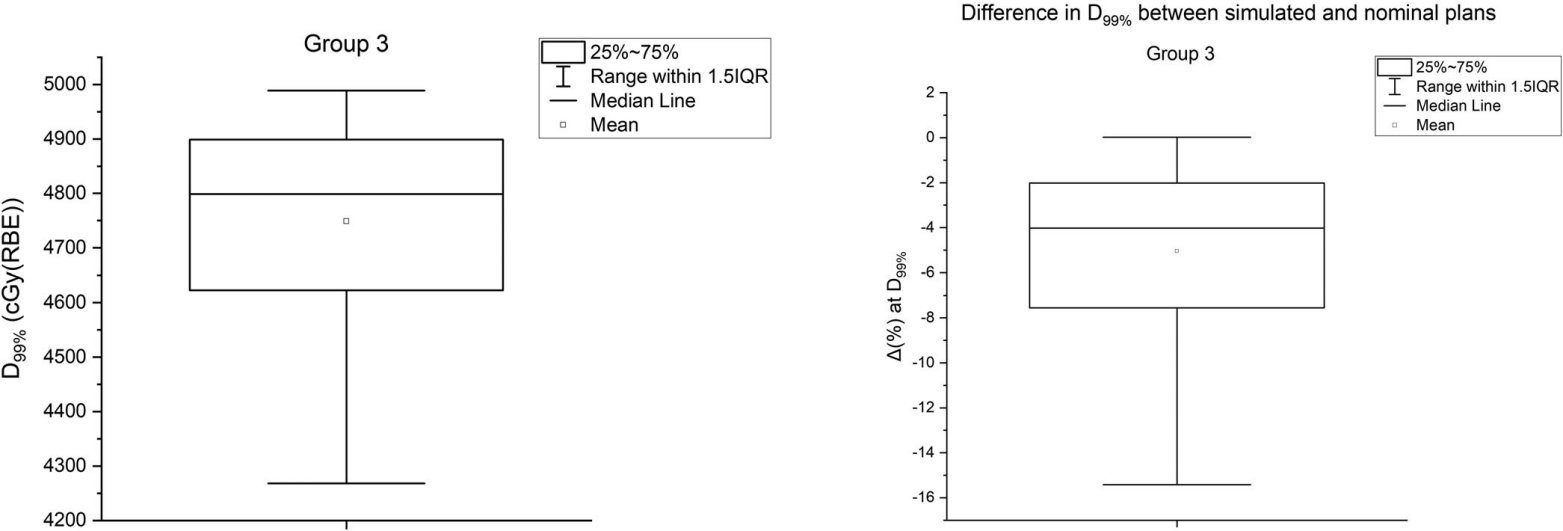
(left panel) The average clinical target volume (CTV) D_99%_ (left panel) from 120 plans of five patients from Group 3 analysis. (right panel) The average difference in CTV D_99%_ between simulated plans in Group 3 and nominal plans. The results are averaged over all five patients.

Figure 4 shows the results for simulated plans when setup (six scenarios) and range (two scenarios) errors are evaluated in conjunction with spot size (±10%) and position (±1 mm) errors occurring simultaneously. The results are based on 160 plans (4 plans for spot size [±10%] and position [±1 mm] errors × 8 scenarios × 5 patients). The average D_99%_ was 4748 cGy(RBE), with an average reduction of 5.0%.

**Fig. 5:**
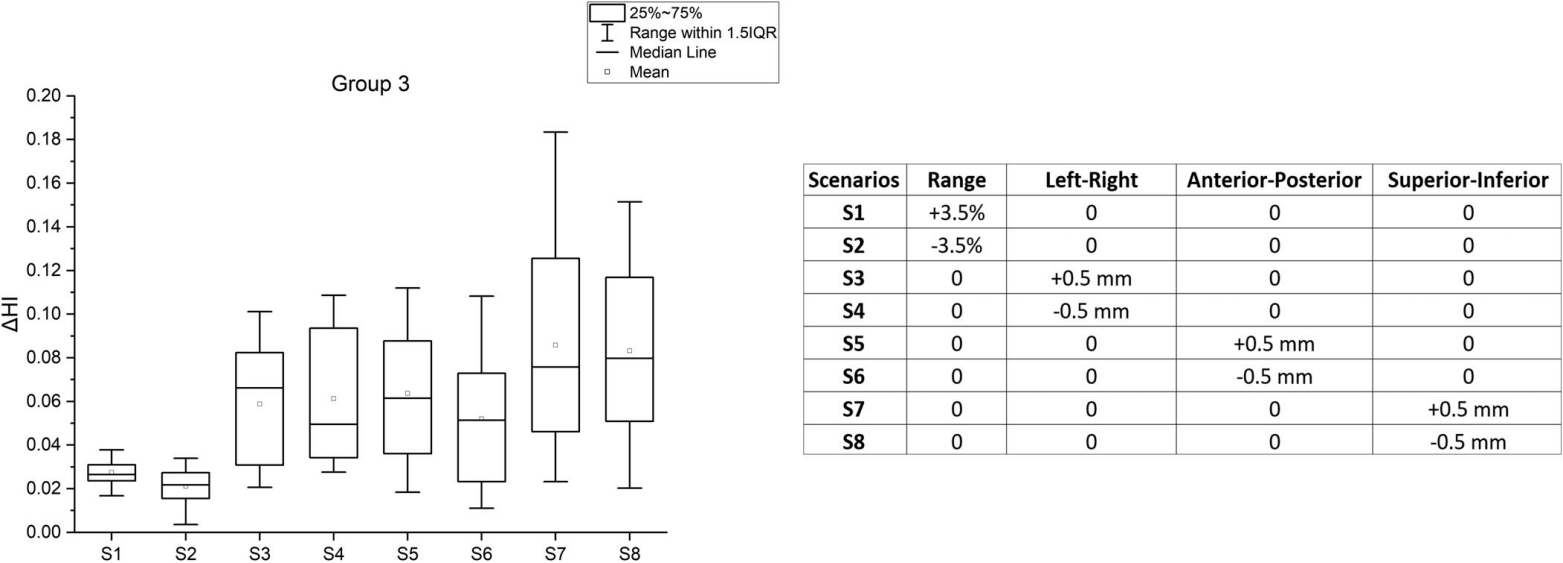
(left panel) The difference in clinical target volume homogeneity index (HI) for eight different scenarios between simulated plans in Group 3 and nominal plans. (right panel) Robustness parameters for eight scenarios of Group 3 analysis. The *D(±10%, ±1 *mm*)* plans are evaluated for setup (±5 mm) and range (±3.5%) uncertainties as described in Section 2.F.

Figure 5 illustrates the difference in HI for various scenarios. The worse HI result was obtained for a 5‐mm isocenter shift in the superior–inferior directions (*y* = ± 5 mm). The average difference in HI was 0.06.

## 4 DISCUSSION

The current study was performed to investigate the dosimetric impact of variations (errors) in the spot sizes and spot positions in PBS proton‐based SBRT lung plans. The results reported in the current study complement previous findings^1^, ^5^, ^7^, ^8^ by adding the impact of variations in spot sizes and positions in robustly optimized PBS lung plans. Due to the availability of the Monte Carlo algorithm in commercially available TPS, researchers are recommending the Monte Carlo algorithm for the optimization and dose calculations in the proton lung plans.^14^, ^15^, ^16^, ^17^ The current study provides additional information regarding the impact of spot size and position errors on the dose distributions of the lung plans, which were robustly optimized (SFO technique) and calculated using the Monte Carlo algorithm.

For a patient cohort in the current study, the variations in spot sizes resulted in decreased target coverage and dose homogeneity. This was true for both the increasing and decreasing spot sizes. For the spot size errors of ±10%, the average loss of target coverage was almost identical. Also, there was a minimum difference in the loss of target coverage between the increasing and decreasing spot sizes for the ±15% category. However, for the spot size errors of ±20%, it was found that the increasing spot sizes resulted in a greater loss of target coverage at D_99%_ when compared with the decreasing spot sizes. The loss of target coverage due to the increasing and decreasing spot sizes can be attributed to the change in the lateral penumbra. The increase in spot size led to the broadening of the lateral penumbra, whereas the decrease in spot sizes led to the contraction of the lateral penumbra.^5^, ^7^ The evaluation at D_1%_ showed that, for the same magnitude of error, the average difference was higher (positive difference) for the decreasing spot sizes than for the increasing spot sizes. These findings suggest that the decreasing spot sizes will also result in overdosage and loss dose homogeneity in the target volume.

During proton beam delivery, there is a probability of variations in both spot sizes and positions. Previous studies^1^, ^2^, ^3^, ^4^, ^5^, ^6^, ^7^, ^8^, ^9^ did not investigate the variations in spot sizes and positions simultaneously but rather focused either on the spot sizes or spot positions. According to AAPM TG224, the recommended tolerances for the spot sizes and spot positions are ±10% and ±1 mm, respectively. By simulating the errors in spot sizes (±10%) and positions (±1 mm) during beam delivery, we quantified the loss of target coverage for a situation when both of these parameters could deviate from the planned parameters. For the decreasing spot sizes (−10%) in conjunction with ±1‐mm spot position errors, the target coverage was reduced by up to 1.1% (at D_99%_). For the increasing spot sizes (+10%) in conjunction with ±1‐mm spot position errors, the target coverage was reduced by up to 2.5% (at D_99%_). If all the results of ±10% spot size errors in conjunction with ±1‐mm spot position errors are analyzed together, the average difference in the target coverage at D_99%_ was −1.3% (range, −0.5% to −2.5%) (Fig. 3). The results from the combined effect of spot size and position errors demonstrated the need for having stringent quality assurance (QA) tolerances to avoid the loss of target coverage due to variations in spot sizes and positions. It is important to note that clinical outcomes can be correlated to the minimum dose to the delivered tumor volume.^23^, ^24^ In a more recent study, Sood et al.^24^ noted the D_99%_ as a potential predictive parameter for clinical outcome in photon‐based lung SBRT.

The majority of the proton centers evaluate the robustness of PBS plans against the setup and range uncertainties,^25^ but there appears to be no common consensus on the plan robustness criteria in the proton therapy community. During PBS proton beam delivery, there is a possibility of delivered spots deviating from their calculated sizes and positions. In the current study, we demonstrated how the variations in spot sizes and positions could be combined with either setup uncertainty or range uncertainty. By assuming the spot size and position errors (±10% and ±1 mm, respectively) occurring simultaneously in conjunction with setup errors, the D_99%_ was decreased by the average difference of 6.1%. Similarly, for the range errors in conjunction with the spot size and spot position errors, we noticed that the average decrease in D_99%_ was by 2.0%. These results suggest that the impact of setup errors was greater by threefold than the impact of range errors in robustly optimized PBS lung plans when spot size and position errors are included in plan robustness evaluation. The spot size and position errors in our study were simulated systematically. During a real clinical scenario of proton beam delivery, the deviations in spot size and position may not be systematic. Future studies should investigate the impact of random occurrence of spot size and position errors in PBS lung cancer plans.

## 5 CONCLUSION

The increasing spot sizes resulted in decreased target coverage and dose homogeneity. Similarly, the decreasing spot sizes led to a loss of target coverage, overdosage, and degradation of dose homogeneity. The addition of spot size and position errors to plan robustness parameters (setup and range uncertainties) increased the target coverage loss and decreased the dose homogeneity.

## CONFLICT OF INTEREST

The authors do not have any relevant conflict of interest to disclose.

## Data Availability

The data that support the findings of this study are available from the corresponding author upon reasonable request.
